# Evisceration Through a Drain Site After Urological Procedures: A Report of Two Cases

**DOI:** 10.7759/cureus.103285

**Published:** 2026-02-09

**Authors:** Sukesh K S, Supreet Verma, Kiran Prabhakar Rebello, Anuj Jain, Deepika H G

**Affiliations:** 1 General Surgery, Autonomous State Medical College, Sultanpur, Sultanpur, IND; 2 General Surgery, King George's Medical University, Lucknow, IND; 3 Surgery, Autonomous State Medical College, Sultanpur, Sultanpur, IND; 4 Anesthesiology, Autonomous State Medical College, Sultanpur, Sultanpur, IND

**Keywords:** abdominal drain, drain site evisceration, evisceration, omentum evisceration, small bowel surgery

## Abstract

Drain placement after abdominal surgery remains a common practice despite ongoing controversy regarding its routine use. Although drains may help detect postoperative bleeding or anastomotic leaks, they can also lead to complications. Drain-site evisceration is a rare but potentially life-threatening complication that requires urgent intervention. Most reported cases occur within a few hours to days following drain removal. We report two unusual cases of drain-site evisceration following urological procedures, with delayed presentation at eight days and 30 days postoperatively. One patient presented with small bowel evisceration requiring emergency laparotomy and bowel resection, while the second patient had omental evisceration managed electively with mesh repair. These cases highlight the importance of early recognition, risk factor identification, and meticulous management of drain-site complications.

## Introduction

Protrusion of organs due to complete or partial separation of a surgical incision is called evisceration [1]. Despite ongoing controversy, placement of drainage systems after abdominal surgery is widely practiced, as it helps prevent the formation of hematoma, seroma, and infected fluid collections; facilitates identification of anastomotic leakage or bleeding; and contributes to the healing process. Common complications include hemorrhage, hollow viscus perforation, infection at the site, fistula formation, kinking and knotting of drains, evisceration, and herniation [2-5]. Here, we report two cases of drain-site evisceration following urological procedures managed at a peripheral tertiary care institute in northern India. The first patient presented with a drain-site evisceration of the small bowel, which required exploratory laparotomy with resection of the diseased bowel. The second patient presented with drain-site evisceration of the omentum, which was managed electively with placement of a preperitoneal mesh.

## Case presentation

Case 1

**Figure 1:**
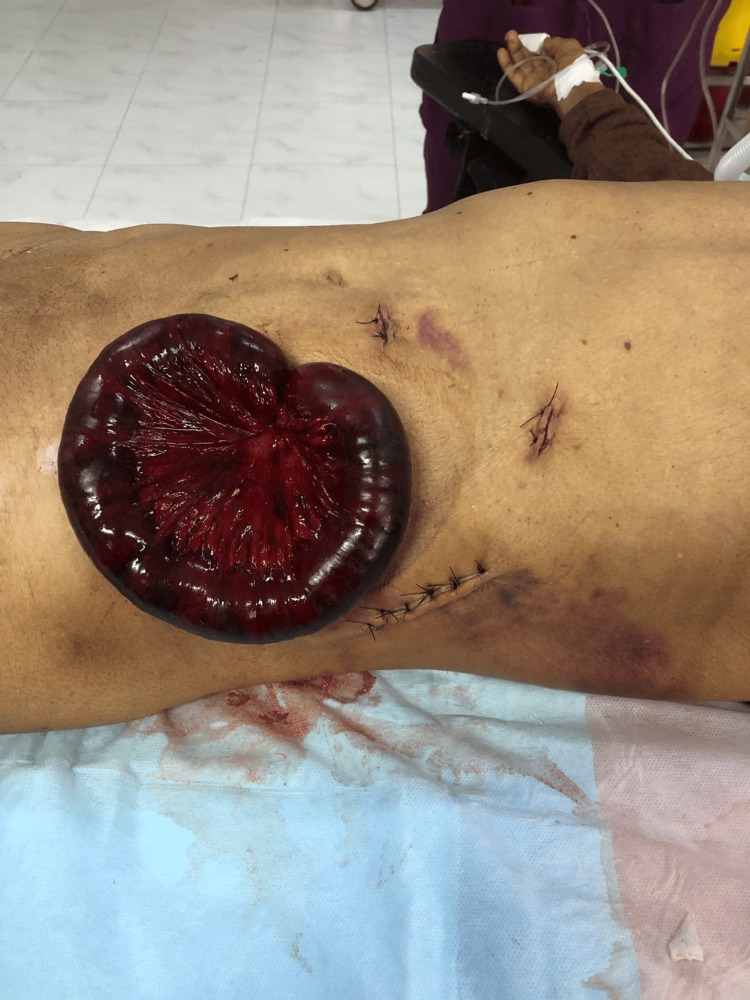
Case 1: Image showing eviscerated small bowel through the drain site and the stitch line of the previous surgery

An 82-year-old male who had undergone laparoscopic radical nephrectomy elsewhere eight days earlier and was discharged on the same day presented to the emergency department with complaints of a mass protruding from the abdominal wall near the drain site after coughing. On examination, the patient was hemodynamically stable, and incarcerated small bowel loops were eviscerating from the drain site in the left upper quadrant, with a healthy stitch line (Figure 1).

**Figure 2:**
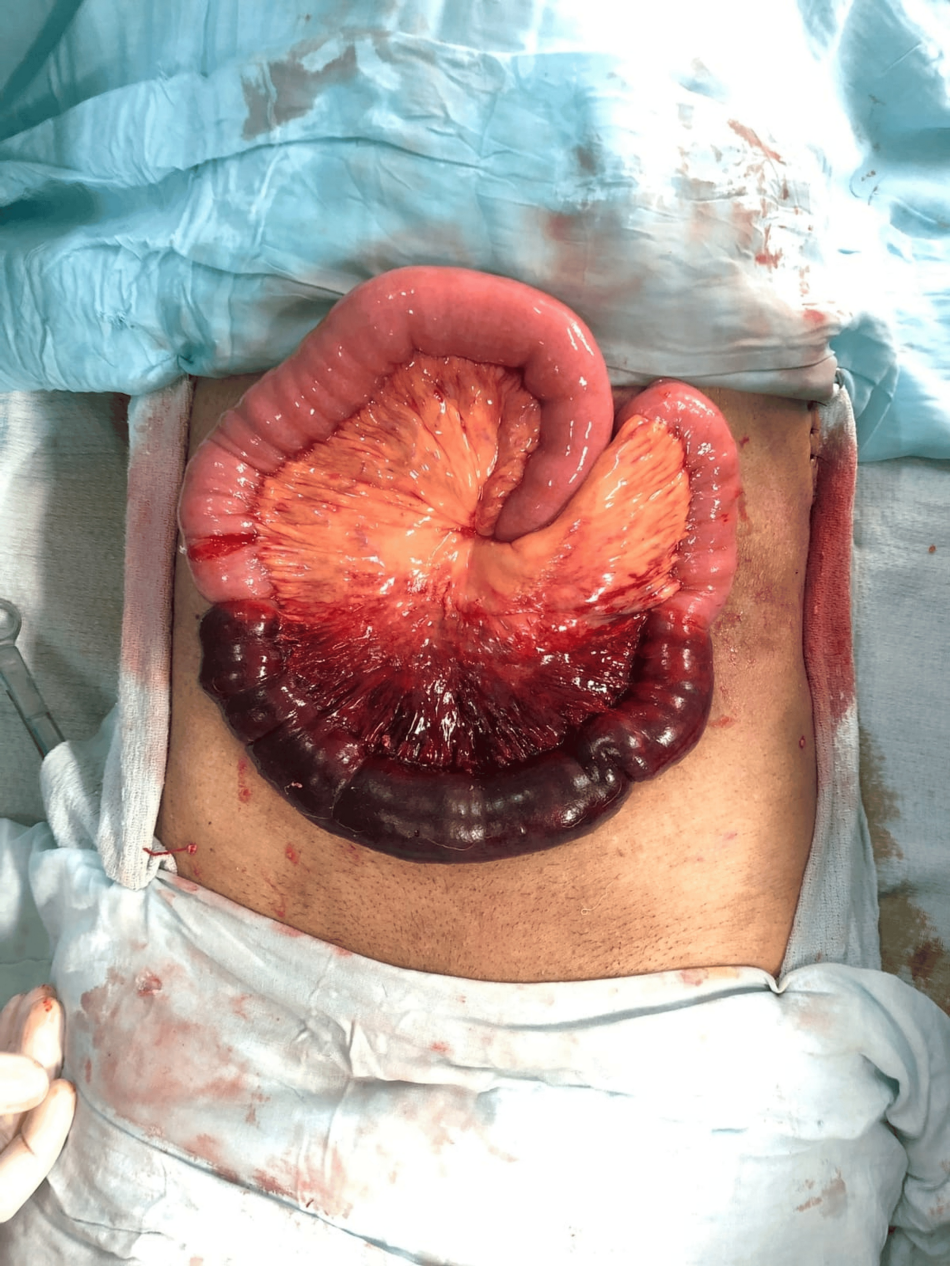
Case 1: Intraoperative image showing diseased small bowel

The patient was rushed to the emergency operating room. Through exploratory laparotomy, the contents were reduced, and resection of 1 foot of gangrenous small bowel (Figure 2), located 3 feet distal to the duodenojejunal junction, was performed, followed by ileoileal anastomosis in an interrupted fashion. The patient had no postoperative complications and was discharged after seven days. The patient was then transferred to the urology institute for further follow-up of the primary surgery.

Case 2

**Figure 3:**
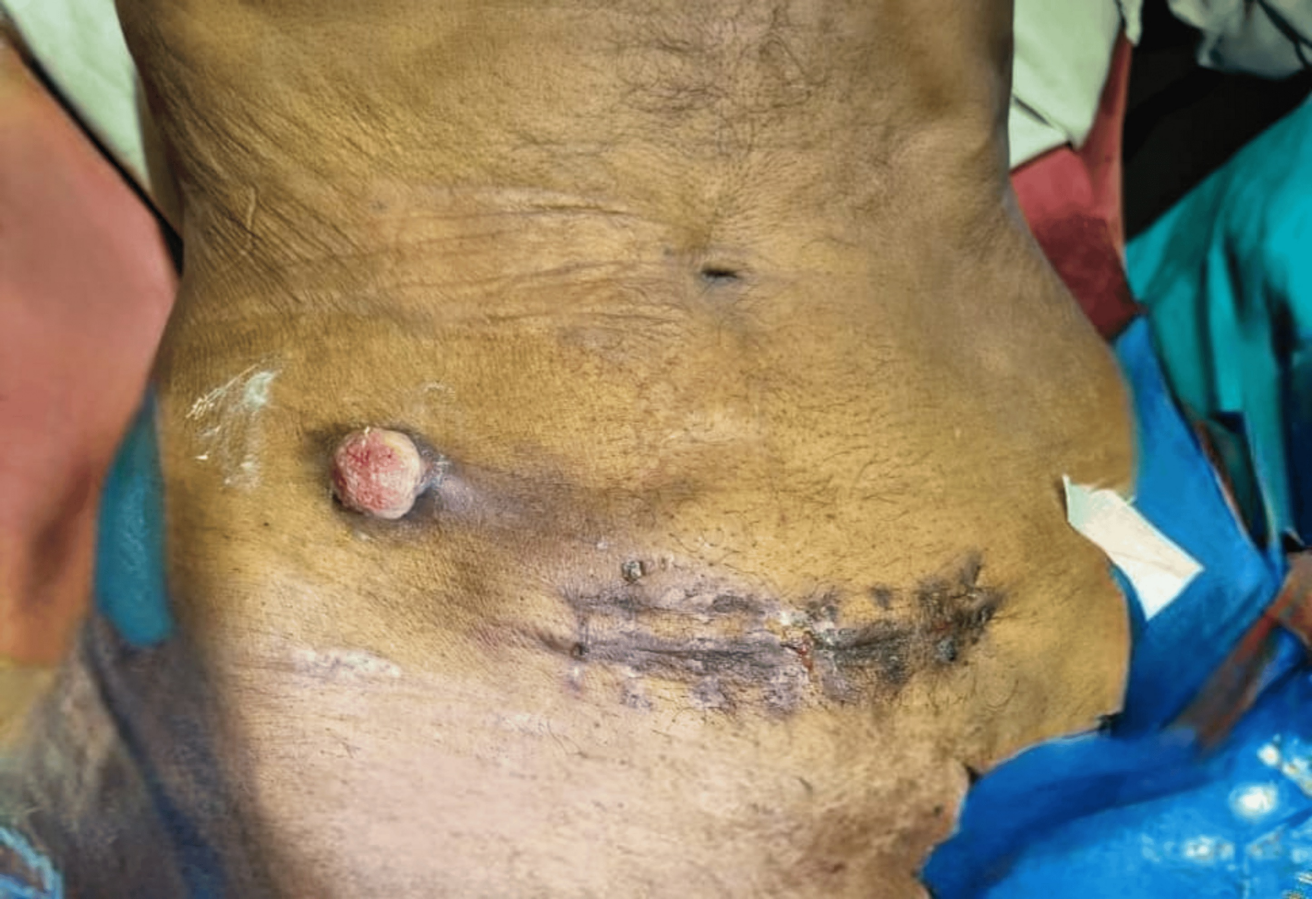
Case 2: Image showing a 2 × 2 cm growth at the drain site with the stitch line of the previous surgery

A 79-year-old male who had undergone open cystolithotomy elsewhere 30 days earlier presented with constant abdominal pain and a mass arising from the drain site, with normal bowel and bladder habits. On examination, a 2 × 2 cm growth with a pinkish hue was present at the drain site, with a healthy stitch line (Figure 3).

**Figure 4:**
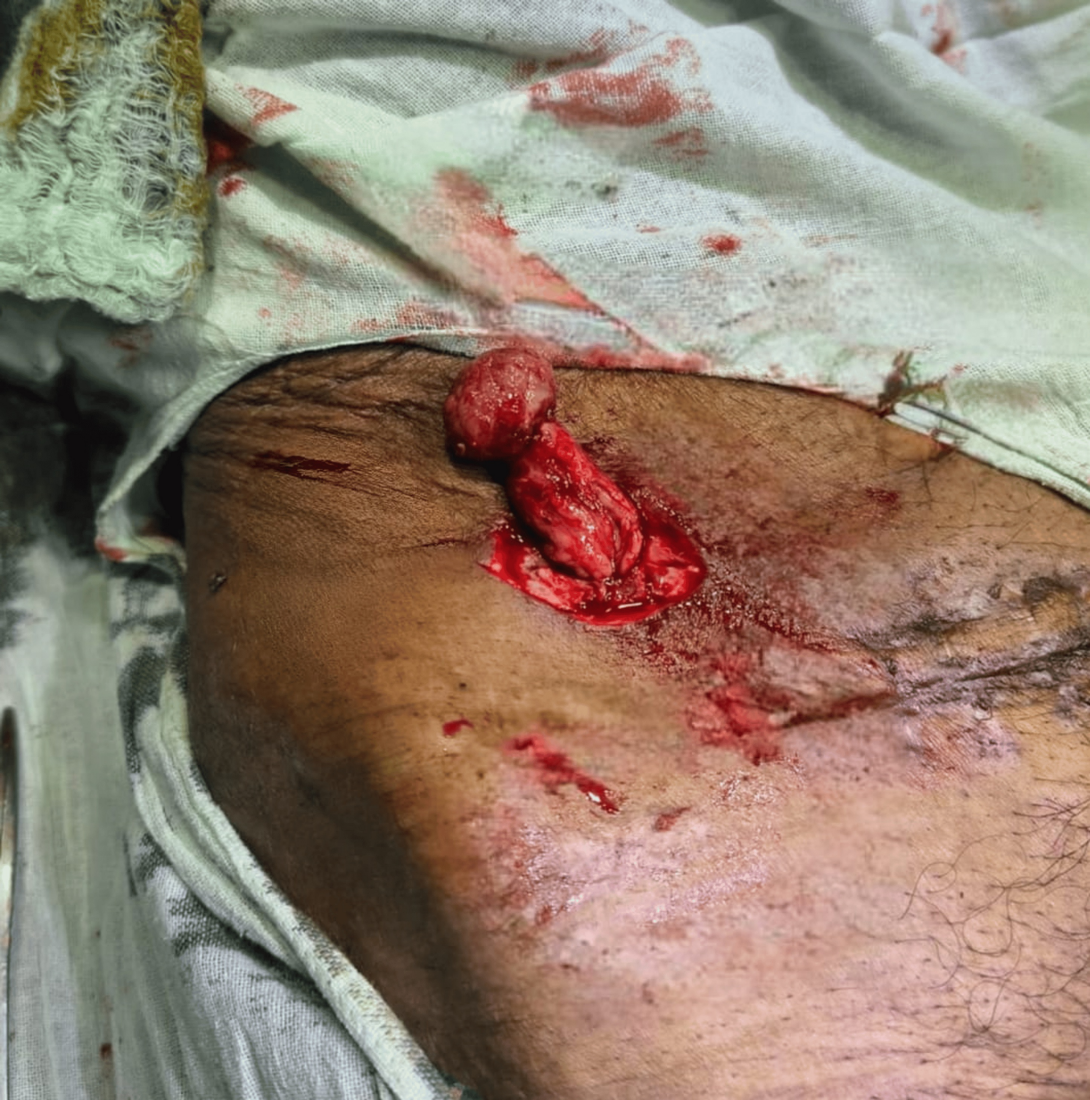
Case 2: Intraoperative image showing eviscerated omentum through the drain site

Cross-sectional imaging revealed a 1-cm defect in the anterior abdominal wall in the right lower quadrant, with herniation of the omentum through it. The patient underwent local site exploration with reduction and resection of the diseased omentum (Figure 4), followed by preperitoneal mesh placement. The postoperative period was uneventful, and the patient was discharged after five days. On follow-up, the patient was doing well with no complaints.

## Discussion

Drain placement after abdominal surgery, despite ongoing controversy, is widely practiced, with advantages including identification of anastomotic leakage or hemorrhage and prevention of seroma or abscess formation [1-5]. Although drain placement is intended to prevent and identify complications, it can itself cause complications such as bleeding, migration, kinking and knotting of drains, drain-site sepsis, fistula formation or erosion into viscera with peritonitis, and evisceration [3,6].

Evisceration, though uncommon, is a known complication that requires emergency intervention. Protrusion of organs due to complete or partial separation of a surgical incision is termed evisceration [1]. Although the small bowel is the most commonly reported organ to eviscerate, cases involving the gallbladder, omentum, appendix, and fallopian tube have also been documented, usually within 24-48 hours after drain removal [2-5]. The former patient presented within 24 hours of drain removal, whereas the latter patient, despite having pain and swelling near the drain site for 15 days, sought medical attention only 30 days after the primary surgery.

Cessation of negative suction and 360° rotation of the drain, which helps break adhesions between the viscera and the drain, are recommended measures to prevent evisceration. Although drain size is a major contributing factor, patient-related factors and surgical technique also contribute. Poor nutritional status, frailty in elderly patients, chronic cough or other causes of increased intra-abdominal pressure, chronic corticosteroid use, and obesity are important patient-related factors. Surgical factors include irregular shape of the drain tract due to multiple attempts at creation, use of large drain tubes with a diameter greater than 1 cm or flat drains, placement of the drain in the midline, and prolonged operative duration [2-7]. Both of our patients were elderly, had a history of cough, and had poor nutritional status, which likely predisposed them to evisceration.

Drain-site evisceration requires immediate intervention, as it poses a risk of strangulation and necrosis of the eviscerated structure. There are reports in which bedside procedures under local anesthesia were performed to reposition the eviscerated organ and avoid imminent complications [3,8].

Papatheodorou et al. reported a case of small bowel evisceration through a drain site following subtotal cholecystectomy for gangrenous ischemic cholecystitis, which required resection and anastomosis, similar to Case 1 [2]. Case 1 involved an elderly patient with poor nutritional status and a history of cough who underwent laparoscopic radical nephrectomy elsewhere and developed drain-site evisceration within 24 hours of drain removal, necessitating emergency laparotomy with resection and anastomosis of the diseased small bowel segment.

Bloom and Ehrlich reported a case of omental evisceration through laparoscopic port sites [9]. Case 2 involved an elderly patient with poor nutritional status and a history of cough who underwent surgery elsewhere for a vesical calculus. The drain was removed on postoperative day 5, and sutures were removed on postoperative day 10 before discharge. The patient complained of pain and a mass at the drain site beginning on postoperative day 15 but sought medical attention only on postoperative day 30, at which time the omentum was found to be eviscerating through the drain site. The patient was managed with local site exploration, reduction and resection of the diseased omentum, and placement of a preperitoneal mesh to reduce the risk of future hernia formation.

## Conclusions

Drain-site evisceration is a rare but serious postoperative complication. Although adherence to meticulous surgical technique and appropriate drain management may reduce the risk, nonmodifiable patient-related factors can make this complication unavoidable. Early diagnosis and timely intervention are crucial to prevent morbidity and mortality.
